# Linkage disequilibrium analysis reveals an albuminuria risk haplotype containing three missense mutations in the cubilin gene with striking differences among European and African ancestry populations

**DOI:** 10.1186/1471-2369-13-142

**Published:** 2012-10-31

**Authors:** Shay Tzur, Walter G Wasser, Saharon Rosset, Karl Skorecki

**Affiliations:** 1 Ruth and Bruce Rappaport Faculty of Medicine and Research Institute, Technion - Israel Institute of Technology, Haifa, 31096, Israel; 2 Division of Nephrology, Rambam Health Care Campus, Haifa, 31096, Israel; 3 Molecular Medicine Laboratory, Rambam Health Care Campus, Haifa, 31096, Israel; 4 Department of Statistics and Operations Research, Tel Aviv University, Tel Aviv, 69978, Israel; 5 8 Ha’Aliyah Street, Haifa, 35254, Israel

## Abstract

**Background:**

A recent meta-analysis described a variant (p.Ile2984Val) in the cubilin gene (CUBN) that is associated with levels of albuminuria in the general population and in diabetics.

**Methods:**

We implemented a Linkage Disequilibrium (LD) search with data from the 1000 Genomes Project, on African and European population genomic sequences.

**Results:**

We found that the p.Ile2984Val variation is part of a larger haplotype in European populations and it is almost absent in west Africans. This haplotype contains 19 single nucleotide polymorphisms (SNPs) in very high LD, three of which are missense mutations (p.Leu2153Phe, p.Ile2984Val, p.Glu3002Gly), and two have not been previously reported. Notably, this European haplotype is absent in west African populations, and the frequency of each individual polymorphism differs significantly in Africans.

**Conclusions:**

Genotyping of these variants in existing African origin sample sets coupled to measurements of urine albumin excretion levels should reveal which is the most likely functional candidate for albuminuria risk. The unique haplotypic structure of CUBN in different populations may leverage the effort to identify the functional variant and to shed light on evolution of the CUBN gene locus.

## Background

Albumin excretion is tightly regulated by the kidney so that under normal circumstances, virtually no albumin appears in the urine [1]. Albuminuria, when present due to kidney disease, is strongly associated with increased risk for cardiovascular disease and mortality [2], as well as progression to end stage kidney disease [3]. Albuminuria is additive to other kidney risk factors such as diabetes and hypertension [2,4,5], and multiple mechanisms for its pathogenesis have been proposed [6,7].

A variant in the cubilin gene (CUBN) that is associated with albuminuria has been recently reported by Boger *et al.*[8]. This gene encodes the cubilin receptor, which is a peripheral membrane protein expressed in the renal proximal tubule [9,10]. It is part of the megalin-cubilin complex receptor, responsible for the reabsorption of albumin [6] and other proteins present in the glomerular ultrafiltrate, minimizing their excretion in healthy human urine [6,10,11]. In addition, cubilin is also responsible for the vital conservation of vitamins and trace elements [10]. Rare mutations in the CUBN gene can cause hereditary megaloblastic anemia and proteinuria (Imerslund-Grasbeck syndrome) [12].

Boger *et al.* reported a statistical association of the common CUBN missense variation p.Ile2984Val (rs1801239, c.8950A>G) with both an elevated urinary albumin-to-creatinine ratio (UACR) (P=1.1x10-11) and with microalbuminuria (P=0.001), in population sample sets including non-diabetic and diabetic subjects [8]. This study reported a large meta-analysis of data from a total of 63,153 individuals of European ancestry and 6,981 African-Americans. The CUBN missense variation p.Ile2984Val was also associated with an increased risk of persistent microalbuminuria in 1,304 patients of European ancestry prospectively followed with Type 1 Diabetes Mellitus (T1DM) , with an estimated hazard ratio per copy of the risk allele of 1.42 (p=0.02) [8]. It should be noted that the allele frequency of this variant according to HapMap [13] is 7.5% in Europeans and 1.8% in west Africans of Yoruba ancestry (YRI).

The recent availability of complete genomic sequence databases allowed us to use a novel approach for searching functional candidate variants that could account for the observed association of the CUBN variant with albuminuria. Our objective was to identify potential functional candidates in the CUBN gene by combining the association that was found in Boger *et al.*[8], with these newly available datasets. By searching data from the 1000 Genomes Project [14], we found that the reported p.Ile2984Val (rs1801239) variant has a frequency close to zero in west Africans, and in Europeans it is actually part of a large intragenic haplotype. This haplotype comprises 19 variants in very high LD, including two additional missense mutations: p.Leu2153Phe (rs62619939) and p.Glu3002Gly (rs1801240). As a result of this high LD, these mutations are expected to yield very similar associations with albuminuria in Europeans. However, in Yoruba, the missense mutations, as well as the other SNPs belonging to the European haplotype, are not in high LD, and have very different allele frequencies. These differences in allele frequencies and LD patterns between different populations could leverage the ability to identify functional variants contributing to different levels of albuminuria.

## Methods

In order to identify SNPs in the CUBN region that are in high LD (with r^2^>0.5) with the SNP reported by Boger *et al.* (rs1801239, p.Ile2984Val) we utilized the 1000 Genomes Project pilot dataset [14] containing the complete genome sequences of 59 Africans from Yoruba (YRI), and 60 individuals of European ancestry from Utah (CEU). The allele frequency of a SNP is calculated by the proportion of one type of allelic variant among the total number of alleles in the sample. We used the program HaploView [15] which provides visualization of LD and haplotype block analysis, to yield the LD values between variants in the region. We identified likely European (CEU) “haplotypes” containing the tagging SNP rs1801239 (p.Ile2984Val), and comprising SNPs that are all in high LD with each other and with this tagging SNP. We then examined the corresponding frequencies of these SNPs and their LD patterns in the YRI sequences, reasoning that differences in LD patterns between the two populations can guide the search for the likely causative variant, underlying the functional association between the gene CUBN and urinary albumin excretion.

## Results and discussion

Of the 3,862 known SNPs in the 306 Kbp of the CUBN gene region, we have identified 18 SNPs that are in very high LD (with r^2^>0.7, and D’=1) with the reported albuminuria associated variant (rs1801239, p.Ile2984Val) in Europeans (Table 1). This combination of 19 SNPs that can be considered as one haplotype, spans 50 Kbp between exons 42 and 57 in the CUBN gene, that contains 67 exons. Among these 19 SNPs is the previously reported variant p.Ile2984Val (c.8950A>G, rs1801239) [8], as well as two additional missense mutations: p.Leu2153Phe (c.6459G>C; rs62619939) and p.Glu3002Gly (c.9005A>G, rs1801240). The other 16 SNPs in this European CUBN haplotype are located within introns remote from intron-exon boundaries. The allele frequency of this haplotype in the European population is about 7%, and therefore according to Hardy-Weinberg equilibrium, 13.5% of the Europeans may carry at least one copy of this risk haplotype. 

**Table 1:** **List of the CUBN SNPs in the extended European Haplotype, including the reported albuminuria risk variant p.Ile2984Val**[8]**and two other missense mutations (p.Leu2153Phe and p.Glu3002Gly)**

**Marker ID**	**Variation Type**	**Allele Frequency YRI (%)**^1^	**Allele Frequency CEU (%)**^1^	**LD with the reported Ile2984Val Variant in CEU population**^1^			**Distance from the reported Ile2984Val Variant (bp)**
				**r^2**	**LOD**	**D’**	
**rs1801240**	**Glu3002Gly**	**21.1**	**6.7**	1.00	10.36	1	−55
**rs1801239**	**Ile2984Val**	**0**^2^	**6.7**^2^	--	--	--	0
rs74440730	Intronic	2.5	5.8	0.87	8.17	1	1,840
rs79051478	Intronic	0	6.7	1.00	10.36	1	5,557
rs78936062	Intronic	1.7	6.7	1.00	10.36	1	8,825
rs74942409	Intronic	0	6.7	1.00	10.36	1	9,249
rs17343073	Intronic	0	6.7	1.00	10.36	1	13,144
rs78625146	Intronic	0	6.7	1.00	10.36	1	14,787
rs79801018	Intronic	9.3	6.7	1.00	10.36	1	18,607
rs78786782	Intronic	9.3	6.7	1.00	10.36	1	18,723
rs73592376	Intronic	50.8	6.7	1.00	10.36	1	18,735
rs77744173	Intronic	0	6.7	1.00	10.36	1	19,219
rs45619139	Intronic	0	6.7	1.00	10.36	1	21,794
rs74375025	Intronic	0	6.7	1.00	10.36	1	28,612
rs45487598	Intronic	0	8.3	0.79	8.18	1	39,601
17006102-Indel ^3^	Intronic indel	15.9	7.1	0.88	8.99	1	47,044
17006421-Indel ^3^	Intronic indel	15.1	7.1	0.88	8.99	1	45,523
**rs62619939**	**Leu2153Phe**	**15.3**	**8.3**	0.79	8.18	1	48,534
rs111263197	Intronic	17.8	8.3	0.79	8.18	1	49,490

^1^ Based on 1000 Genomes Project pilot study, CEU n=120, YRI n=118.

^2^ In larger dataset of HAPMAP (CEU n=226, YRI n=226), the estimated minor allele frequency of rs1801239 was 7.5% in CEU and 1.8% in YRI.

^3^ This indel (insertion/deletion) variant was reported by 1000 Genomes Project pilot study, but has not been reported elsewhere as yet.

This extended European haplotype was not present among Yoruba (YRI), as might be expected given the longer recombination history in Africans [16,17]. An analysis of the YRI haplotypes in the 1000 Genomes database revealed significantly different minor allele frequencies of the missense variants: p.Ile2984Val (rs1801239), 6.7% in CEU, 0% in YRI; p.Glu3002Gly (rs1801240), 6.7% in CEU, 21.2% in YRI; and p.Leu2153Phe (rs62619939), 8.3% in CEU, 15.3% in YRI. We successfully validated the African ancestry allele frequency differences of the variants p.Ile2984Val and p.Glu3002Gly by PCR and Sanger sequencing in 26 healthy African Americans individuals, and found minor allele frequencies similar to those computed from the 1000 Genomes Project (p.Ile2984Val =0%, p.Glu3002Gly =28.8%) (PCR conditions are available upon request). The minor allele frequency of the p.Ile2984Val variant reported as being associated with albuminuria is between 0–1.8% in African ancestry populations, according to 1000 Genomes Project, HapMap and our own genotyping. This low frequency in west Africans raises doubts as to whether the attributable albuminuria risk found for this variant in Europeans (allele frequency of 6.7%) can explain the same risk found in African ancestry populations (allele frequency of ~0%).

Although African Americans were included in the meta-analysis of Boger *et al.*, the results in that publication cannot be used to determine which of the missense mutations demonstrates association in African ancestry populations and thereby is possibly more likely to be functional, since only one (p.Ile2984Val) of the three missense variants highlighted in our analysis was actually tested in the Boger et al. study [8]. Moreover, because African Americans, unlike continental African individuals, also have about 20% European ancestry [17], some of the risk variants in this population might be of European origin.

Interestingly, if the reported p.Ile2984Val variant [8] is not causative, then we may expect the low value of 0.15% estimated for percentage of UACR variance explained by this variant in Europeans to underestimate the actual percentage variance explained by the most highly associated of the relevant variants in African and African American populations [8]. In YRI 1000 Genomes Project samples, allele frequencies of the risk variants for the SNPs rs1801240 (p.Glu3002Gly) and rs1801239 (p.Ile2984Val) are 21.2% and 0% (1.8% according to HapMap), respectively, compared to 6.7% for both SNPs in Europeans. This leads to an estimated 0.4% variance of UACR explained in Yoruba if rs1801240 (p.Glu3002Gly) is albuminuria risk causative, and an order of magnitude lower expected value of 0.04% or less if rs1801239 (p.Ile2984Val) is risk causative (This calculation is based on the general formula for variance explained in linear regression: R^2^ = β^2^ * var(X) / var(Y). So if var(X) is increased from 0.067*0.93=0.062 to 0.212*0.788 =0.167 and beta and var(Y) remain fixed, then R^2^ grows from 0.15% to 0.4%). Therefore, we can suggest that differences in allele frequencies and LD patterns between Africans and Europeans can be used in future association studies in Africans or African Americans, in which such newly appreciated candidate SNPs would be directly genotyped to inform as to which are the more likely candidate(s) for functional risk causation.

The mutations p.Ile2984Val and p.Glu3002Gly are located in exon 57 in a region that is part of the 22nd CUB domains, out of a total of 27 domains in cubilin that confer binding ability to a variety of ligands [10]. *In vitro* experiments showed that CUB domains 22 through 27 demonstrated calcium dependent binding to megalin [18]. The third missense mutation in exon 42, p.Leu2153Phe, is thought to be in the region of CUB domain 15, which is adjacent to CUB domains 13–14 that are considered to be involved in a receptor associated protein-binding site [19]. The presence of an extended haplotype of 50 kbp in Europeans may conceivably reflect the effect of a selective sweep due to positive selection pressure under vitamin B12 or other nutritional influences during human evolution [20,21]. It could be speculated that a particular variant in this European haplotype might be involved in nutritional selective advantage or other adaptations during human evolution and migration. According to PolyPhen2 [22], the mutations p.Ile2984Val and p.Glu3002Gly are predicted not to alter protein function, while the missense mutation p.Leu2153Phe is predicted to impair protein function. Similar programs, such as SIFT [23] and MutationTaster [24]**,** designated the three variants as tolerated polymorphisms.

The main limitation of the present study is the lack of available samples to test the real association of the suggested candidate variants, since the sample size that is needed is large, while the effect of the variant is small. In addition, it is also possible that a variant in a non-coding region is responsible to the effect found by Boger et al. [8]. Another possible limitation is that we based our analysis on publically available sequences, and deep sequencing methods have limitations related to alignment problems and base calling. It is also should be recalled that association is not causation, and only biological studies can actually prove functionality, especially given the complexity of albuminuria and its relation to both the glomerular filtration and tubular reabsorption.

The reported association for the CUBN variant, p.Ile2984Val, led Boger *et al.* to suggest that levels of albuminuria in the general population are determined by tubular reabsorption acting in concert with glomerular filtration [8]. The finding of a functional variant, which alters protein function, would greatly enhance our understanding of the mechanisms of albumin excretion in kidney health and disease. Moreover, we have presented a generic data searching and computational approach that is based on data mining, bioinformatic and population leveraging, which can spawn a wave of association studies following up on Genome Wide Association Studies (GWAS) results. A similar approach was successfully implemented in previous studies in which variants in the APOL1 gene were found to be highly associated with non- diabetic end stage kidney disease in African Americans [25,26]. Future follow up studies can use existing well-phenotyped sample sets, with a relatively small number of genetic tests, and attain far greater statistical power, by utilizing population ancestry differences in genetic architecture (LD patterns, allele frequencies, and population leveraging). This approach can also be applied to associations reported in other GWAS in common renal and non-renal disease phenotypes.

## Conclusion

The current study identified a haplotype in the CUBN gene that exists only in Europeans and contains three missense mutations, of which one is the variant p.Ile2984Val associated with levels of albuminuria. These three mutations (p.Glu3002Gly; p.Ile2984Val; p.Leu2153Phe) have different LD pattern and allele frequencies in west Africans, and we suggest using this population to evaluate which of the three are most likely to be functional. Identifying the true causative has significant implications for predicting albuminuria-related risks in African-ancestry individuals and in the general population. Studying the genetic basis of these variants may also shed light on evolution of the CUBN gene locus with respect to human evolutionary history.

## Competing interests

The authors declare that they have no competing interests.

## Authors’ contributions

ST participated in the design of the study and drafted the manuscript. SR participated in the design of the study and carried out the statistical analysis. WW drafted the manuscript and participated in the design of the study. KS conceived of the study and participated in its design and coordination, and also drafted the manuscript. All authors read and approved the final manuscript.

## Pre-publication history

The pre-publication history for this paper can be accessed here:

http://www.biomedcentral.com/1471-2369/13/142/prepub
